# A Novel Evidence Theory and Fuzzy Preference Approach-Based Multi-Sensor Data Fusion Technique for Fault Diagnosis

**DOI:** 10.3390/s17112504

**Published:** 2017-10-31

**Authors:** Fuyuan Xiao

**Affiliations:** School of Computer and Information Science, Southwest University, No. 2 Tiansheng Road, BeiBei District, Chongqing 400715, China; xiaofuyuan@swu.edu.cn

**Keywords:** sensor data fusion, evidential conflict, evidence distance, belief entropy, variance of entropy, fuzzy preference relations, Dempster–Shafer evidence theory, fault diagnosis

## Abstract

The multi-sensor data fusion technique plays a significant role in fault diagnosis and in a variety of such applications, and the Dempster–Shafer evidence theory is employed to improve the system performance; whereas, it may generate a counter-intuitive result when the pieces of evidence highly conflict with each other. To handle this problem, a novel multi-sensor data fusion approach on the basis of the distance of evidence, belief entropy and fuzzy preference relation analysis is proposed. A function of evidence distance is first leveraged to measure the conflict degree among the pieces of evidence; thus, the support degree can be obtained to represent the reliability of the evidence. Next, the uncertainty of each piece of evidence is measured by means of the belief entropy. Based on the quantitative uncertainty measured above, the fuzzy preference relations are applied to represent the relative credibility preference of the evidence. Afterwards, the support degree of each piece of evidence is adjusted by taking advantage of the relative credibility preference of the evidence that can be utilized to generate an appropriate weight with respect to each piece of evidence. Finally, the modified weights of the evidence are adopted to adjust the bodies of the evidence in the advance of utilizing Dempster’s combination rule. A numerical example and a practical application in fault diagnosis are used as illustrations to demonstrate that the proposal is reasonable and efficient in the management of conflict and fault diagnosis.

## 1. Introduction

The multi-sensor data fusion technique plays a significant role in fault diagnosis. Due to the complexity of the targets, the report collected from a single sensor is insufficient in decision making processes. Additionally, because of the impact of the environment, the data gathered from multiple sensors may be unreliable or even wrong so that it can cause erroneous results in fault diagnosis. Hence, multi-sensor data fusion technologies are required in various fields of practical applications [1,2,3,4,5,6,7,8,9], especially in the area of data fusion using vibration data [10,11,12,13,14,15]. However, in the practical applications, the data that are gathered from the multi-sensors are usually uncertain. An open issue is how to model and handle such kinds of uncertain information. To address this issue, a variety of theoretical methods has been exploited for multi-sensor data fusion, like the rough sets theory [16,17], fuzzy sets theory [18,19,20,21,22], evidence theory [23,24,25], Z numbers [26,27], and D numbers theory [28,29,30], evidential reasoning [31,32,33,34], and so on [35,36,37,38].

Dempster–Shafer evidence theory, which is an uncertainty reasoning tool, was firstly proposed by Dempster [23]; then, it was developed by Shafer [24]. Because Dempster–Shafer evidence theory is flexible and effective in modeling the uncertainty regardless of prior information, it is widely applied to various areas of information fusion, like pattern recognition [39,40,41], decision making [42,43,44,45,46,47], supplier selection [48,49], optimization problems [50,51], risk analysis [52,53,54] and fault diagnosis [55,56,57,58,59,60]. Although Dempster–Shafer evidence theory has many advantages, it may generate counter-intuitive results, when fusing highly conflicting pieces of evidence [61,62]. To solve this problem, many methods have been proposed. They are divided into two types of methodologies [63,64,65,66,67]. The first type involves modifying Dempster’s combination rule, while the second type involves pre-processing the bodies of evidence. The main research works for the first type include the unnormalized combination rule presented by Smets [68], the disjunctive combination rule proposed by Dubois and Prade [69] and the combination rule presented by Yager [70]. Nevertheless, the modification of the combination rule often destructs the good properties, like the commutativity and associativity. Furthermore, if sensor failure results in the counter-intuitive results, such a modification is regarded to be unreasonable. Therefore, many researchers pre-process the bodies of evidence to resolve the problem of highly conflicting evidence, which falls into the second type. The main research works for the second type include the simple average approach of the bodies of evidence proposed by Murphy [71], the weighted average of the masses based on the evidence distance presented by Deng et al. [72] and the cosine theorem-based method proposed by Zhang et al. [73]. Deng et al.’s weighted average approach [72] overcomes the weakness of Murphy’s method [71] to some extent. Later on, Zhang et al. [73] made an improvement based on [72] and introduced the concept of vector space to handle the conflicting evidence. However, the effect of evidence’s uncertainty itself on the weight was overlooked.

In this paper, therefore, a novel multi-sensor data fusion method is proposed, which is a hybrid methodology in terms of the distance of evidence, belief entropy and fuzzy preference relation analysis. The proposal considers the support degree among the pieces of evidence, the uncertainty measure of the evidence and the effect of the relative credibility of the evidence on the weight, so that it can obtain more appropriately weighted average evidence before using Dempster’s combination rule. Specifically, the proposed method consists of the following procedures. First, in order to measure the support degree between the pieces of evidence, the function of evidence distance is leveraged, where the support degree represents the reliability of the evidence. After that, the relative credibility preference of the evidence is indicated by taking advantage of the fuzzy preference relation analysis on the foundation of the uncertainty of each piece of evidence measured by the belief entropy. Based on that, the support degrees of the evidence are adjusted, which can be utilized to generate the appropriate weights with regard to the evidence. Finally, the weighted average evidence can be obtained on the basis of the modified weights of the evidence before using Dempster’s combination rule. A numerical example and a practical application in fault diagnosis are used as illustrations to demonstrate that the proposed method outperforms the related methods with respect to the conflict management and fault diagnosis.

The remaining content of this paper is arranged below. Section 2 introduces the preliminaries of this paper briefly. In Section 3, a novel multi-sensor data fusion approach with regard to fault diagnosis is proposed. Section 4 gives a numerical example to illustrate the effectiveness of the proposal. Then, the proposed method is applied to a practical application in fault diagnosis in Section 5. Finally, Section 6 gives the conclusion.

## 2. Preliminaries

### 2.1. Dempster–Shafer Evidence Theory

Dempster–Shafer evidence theory [23,24] is extensively applied to handle uncertain information that belongs to the category of artificial intelligence. Because Dempster–Shafer evidence theory is flexible and effective in modeling the uncertainty regardless of prior information, it requires weaker conditions compared with the Bayesian theory of probability. When the probability is confirmed, Dempster–Shafer evidence theory degenerates to the probability theory and is considered as a generalization of Bayesian inference [74]. In addition, Dempster–Shafer evidence theory has the advantage that it can directly express the “uncertainty” via allocating the probability into the set’s subsets, which consists of multi-objects, instead of a single object. Furthermore, it is capable of combining the bodies of evidence to derive new evidence. The basic concepts and definitions are described as below.

**Definition** **1** (Frame of discernment)**.** *Let* Θ *be a nonempty set of events that are mutually-exclusive and collectively-exhaustive, defined by:*

(1) $$\Theta =\{F_{1},F_{2},\ldots ,F_{i},\ldots ,F_{N}\},$$

*in which the set* Θ *denotes a frame of discernment.*

*The power set of* Θ *is represented as $2^{\Theta }$, where:*

(2) $$2^{\Theta }=\left\{\emptyset ,\left\{F_{1}\right\},\left\{F_{2}\right\},\ldots ,\left\{F_{N}\right\},\left\{F_{1},F_{2}\right\},\ldots ,\left\{F_{1},F_{2},\ldots ,F_{i}\right\},\ldots ,\Theta \right\},$$

*and* ∅ *is an empty set.*

*When A is an element of the power set of* Θ, *i.e., $A\in 2^{\Theta }$, A is called a hypothesis or proposition.*

**Definition** **2** (Mass function)**.** *In the frame of discernment* Θ, *a mass function m is represented as a mapping from $2^{\Theta }$ to [0, 1] that is defined as:*

(3) $$m:\;2^{\Theta }\to \left[0,1\right],$$

*which meets the conditions below:*

(4) $$\begin{array}{cc} m(\emptyset ) & =0,\\ \sum_{A\in 2^{\Theta }}m\left(A\right) & =1. \end{array}$$

The mass function *m* in the Dempster–Shafer evidence theory can also be called a basic probability assignment (BPA). When m(A) is greater than zero, *A* as the element of $2^{\Theta }$ is named as a focal element of the mass function, where the mass function m(A) indicates how strongly the evidence supports the proposition or hypothesis *A*.

**Definition** **3** (Belief function)**.** *Let A be a proposition where A⊆Θ; the belief function Bel of the proposition A is defined by:*

(5) $$\begin{array}{cc} Bel:\; & 2^{\Theta }\to \left[0,1\right],\\ & Bel\left(A\right)=\sum_{B\subseteq A}m\left(B\right). \end{array}$$

*The plausibility function Pl of the proposition A is defined by:*

(6) $$\begin{array}{cc} Pl:\; & 2^{\Theta }\to \left[0,1\right],\\ & Pl\left(A\right)=1-Bel\left(\bar{A}\right)=\sum_{B\cap A\neq \emptyset }m\left(B\right), \end{array}$$

*where $\bar{A}$ is the complement of A, such that $\bar{A}=\Theta -A$.*

Apparently, the plausibility function Pl(A) is equal to or greater than the belief function Bel(A), where the belief function Bel is the lower limit function of the proposition *A*, and the plausibility function Pl is the upper limit function of the proposition *A*.

**Definition** **4** (Dempster’s rule of combination)**.** *Let two basic probability assignments (BPAs) be $m_{1}$ and $m_{2}$ on the frame of discernment* Θ *where the BPAs $m_{1}$ and $m_{2}$ are independent; Dempster’s rule of combination, defined by $m=m_{1}⊕m_{2}$, which is called the orthogonal sum, is represented as below:*

(7) $$m\left(A\right)=\left\{\begin{array}{ll} \frac{1}{1-K}\sum_{B\cap C=A}m_{1}\left(B\right)m_{2}\left(C\right), & A\neq \emptyset ,\\ 0, & A=\emptyset , \end{array}\right.$$

*with:*

(8) $$K=\sum_{B\cap C=\emptyset }m_{1}\left(B\right)m_{2}\left(C\right),$$

*where B and C are also the elements of $2^{\Theta }$ and K is a constant that presents the conflict between the BPAs $m_{1}$ and $m_{2}$.*

Notice that Dempster’s combination rule is only practicable for the BPAs $m_{1}$ and $m_{2}$ under the condition that K<1.

### 2.2. Distance of Pieces of Evidence

Jousselme et al. [75] presented a distance function of the evidence to measure the distance among the basic probability assignments (BPAs), which is defined as below.

**Definition** **5** (Distance between two BPAs)**.** *Let two basic probability assignments (BPAs) $m_{1}$ and $m_{2}$ be on the same frame of discernment* Θ, *which contains N number of mutually-exclusive and collectively-exhaustive propositions. The distance between the BPAs $m_{1}$ and $m_{2}$ is denoted as:*

(9) $$d\left(m_{1},m_{2}\right)=\sqrt{\frac{1}{2}{\left(\vec{m_{1}}-\vec{m_{2}}\right)}^{T}\underset{=}{D}\left(\vec{m_{1}}-\vec{m_{2}}\right)},$$

*where $\vec{m_{i}}\left(i=1,2\right)$ is a $2^{N}$-dimensional column vector and $\underset{=}{D}$ is a $\left(2^{N}\times 2^{N}\right)$-dimensional matrix.*

*The elements of $\underset{=}{D}$ that measure the conflict of the focal elements in the BPAs $m_{1}$ and $m_{2}$ can be represented as:*

(10) $$\underset{=}{D}\left(A,B\right)=\frac{\left|A\cap B\right|}{\left|A\cup B\right|},$$

*where $A\in 2^{\Theta }$ and $B\in 2^{\Theta }$. $\left|A\cap B\right|$ denotes the amount of objects in common between the elements A and B, while $\left|A\cup B\right|$ represents the subset’s cardinality of the union elements A and B.*

It can be stated that $\underset{=}{D}\left(A,B\right)\in \left[0,1\right]$. Specifically, the value of $\left|A\cap B\right|$ is zero, when no common items exist between the elements *A* and *B*, which means that the element *A* highly conflicts with the element *B* so that the degree of similarity between the elements *A* and *B* is zero. Therefore, the smaller the $\underset{=}{D}\left(A,B\right)$ is, the less similarity between the elements *A* and *B* there is; whereas, $\underset{=}{D}\left(A,B\right)=1$ indicates that the element *A* is identical to the element *B*.

### 2.3. Belief Entropy

A belief entropy, called the Deng entropy, was first proposed by Deng [43] and has been applied in various fields [76]. As the generalization of the Shannon entropy [77,78], the Deng entropy is an effective math tool for measuring the uncertain information, because the uncertain information can be expressed by BPAs, so that it can be used in the evidence theory. In such a situation that the uncertainty is expressed by the probability distribution, the uncertain degree measured by the Deng entropy will be identical to the uncertain degree measured by the Shannon entropy. The basic concepts and definitions are introduced below.

Let *A* be a proposition of the basic probability assignment (BPA) *m* on the frame of discernment Θ; the Deng entropy $E_{d}\left(m\right)$ of the BPA *m* is defined as follows:

(11) $$E_{d}\left(m\right)=-\sum_{A\subseteq \Theta }m\left(A\right)\;log\;\frac{m(A)}{2^{\left|A\right|}-1},$$

where |A| is the cardinality of the proposition *A*.

When the belief is only allocated to the single object, which means that |A|=1, the Deng entropy degenerates to the Shannon entropy, namely,

(12) $$E_{d}\left(m\right)=-\sum_{A\in \Theta }m\left(A\right)\;log\;\frac{m(A)}{2^{\left|A\right|}-1}=-\sum_{A\in \Theta }m\left(A\right)\;log\;m\left(A\right).$$

The larger the cardinality of the proposition is, the larger the Deng entropy of evidence is, so that the evidence contains more information. When a piece of evidence has a big Deng entropy, it is supposed to be better supported by other evidence, which represents that this evidence plays an important part in the final combination.

### 2.4. Fuzzy Preference Relations

Fuzzy preference relations play a fundamental part in many decision-making processes, and they were first presented by Tanino [79] in 1984. It is a kind of method that can construct the decision matrices of pairwise comparisons by using the linguistic values that are provided by experts. The basic concepts are introduced below.

**Definition** **6** (Fuzzy preference relations [79,80,81,82])**.** *Let P be a fuzzy preference relation and $X=\{A_{1},A_{2},\ldots ,A_{k}\}$ be a set of alternatives, where X⊆Θ, then the fuzzy preference relation is defined as follows:*

(13) $$P={\left(p_{ij}\right)}_{k\times k}=\left[\begin{array}{ccccc} 0.5 & \cdots & p_{1i} & \cdots & p_{1k}\\ \vdots & \vdots & \vdots & \vdots & \vdots \\ p_{i1} & \cdots & 0.5 & \cdots & p_{ik}\\ \vdots & \vdots & \vdots & \vdots & \vdots \\ p_{k1} & \cdots & p_{ki} & \cdots & 0.5 \end{array}\right],\;$$

*where $p_{ij}\in \left[0,1\right]$ (1≤i≠j≤k) represents the preference value for the alternative $A_{i}$ over $A_{j}$, which meets the conditions below:*

(14) $$p_{ij}+p_{ji}=1\;and\;p_{ii}=0.5.$$

It should be stated that $p_{ij}=0.5$ represents the indifference between the alternatives $A_{i}$ and $A_{j}$; $p_{ij}=1$ represents that $A_{i}$ is absolutely preferred by $A_{j}$; $p_{ij}>0.5$ represents that $A_{i}$ is preferred by $A_{j}$.

Whereas, the preference values may be inconsistent in the fuzzy preference relation, hence, Tanino [79] proposed the concept of the additive consistency for the fuzzy preference relation $P={\left(p_{ij}\right)}_{k\times k}$ as follows:

(15) $$p_{ir}=p_{ij}+p_{jr}-0.5,$$

where $p_{ii}=0.5$ and $p_{ij}+p_{ji}=1$ (1≤i≠j≠r≤k).

After that, Lee [80] claimed that the complete fuzzy preference relation may not satisfy the consistency of the order in certain cases. Hence, the consistency of the order in fuzzy preference relations was presented by Lee [80] to solve this problem.

**Definition** **7** (The consistency matrix [80])**.** *Let $P^{*}={\left(p_{ij}\right)}_{k\times k}$ be a complete fuzzy preference relation, in which $p_{ij}$ represents the preference values for the alternative $A_{i}$ over $A_{j}$, $p_{ij}+p_{ji}=1$ and $p_{ii}=0.5$ (1≤i≠j≤k). The consistency matrix $\bar{P}$ can be constructed on the basis of the complete fuzzy preference relation $P^{*}$, which is defined by:*

(16) $$\bar{P}={\left({\bar{p}}_{ir}\right)}_{k\times k}={\left(\frac{1}{k}\sum_{j=1}^{k}\left(p_{ij}+p_{jr}\right)-0.5\right)}_{k\times k}.$$

The consistency matrix $\bar{P}={\left({\bar{p}}_{ir}\right)}_{k\times k}$ (1≤i≠r≤k) has the properties below:

(1) ${\bar{p}}_{ir}+{\bar{p}}_{ri}=1$;
(2) ${\bar{p}}_{ii}=0.5$;
(3) ${\bar{p}}_{ir}={\bar{p}}_{ij}+{\bar{p}}_{jr}-0.5$;
(4) ${\bar{p}}_{ir}\leq {\bar{p}}_{is}$ for all i∈{1,2,…,k}, where s≠i and s≠r.

Let $\bar{P}={\left({\bar{p}}_{ir}\right)}_{k\times k}$ be a consistency matrix; the ranking value of alternative $A_{i}$, denoted as $RV(A_{i})$, is defined by:

(17) $$RV\left(A_{i}\right)=\frac{2}{k^{2}}\sum_{j=1}^{k}{\bar{p}}_{ij},$$

where 1≤i≤k and $\sum_{i=1}^{k}RV\left(A_{i}\right)=1$.

## 3. The Proposed Method

In this paper, by considering not only the conflicts between pieces of evidence, but also the impact of the evidence’s uncertainty itself, a novel multi-sensor data fusion approach is presented and applied in fault diagnosis. The proposed method is a hybrid methodology that integrates the distance of evidence, belief entropy and fuzzy preference relation analysis, which consists of the following parts.

**Figure 1:**
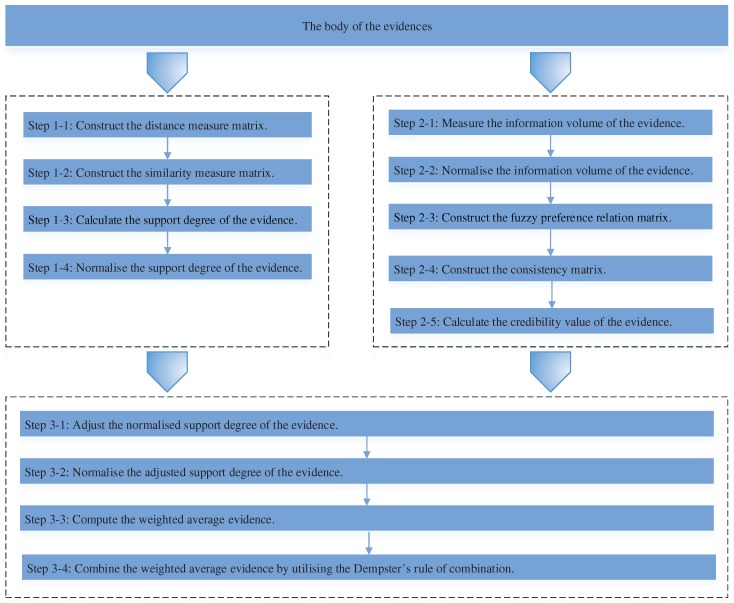
The flowchart of the proposed method.

The function of evidence distance is first leveraged for measuring the conflict degree among the pieces of evidence, then the support degree resulting from the distance of the evidence is obtained to denote the evidence’s reliability. When the evidence is well supported by other pieces of evidence, it is supposed to have less conflict with other pieces of evidence, so that a big weight should be allocated to this piece of evidence. Instead, when the evidence is poorly supported by other pieces of evidence, it is regarded to highly conflict with other pieces of evidence so that a small weight should be allocated to this evidence. Next, the information volume of the evidence is calculated by making use of the belief entropy. Based on the calculated quantitative information volume, the fuzzy preference relations analysis is applied to indicate the relative credibility preference in terms of the pieces of evidence. Whereafter, the support degree of the evidence is adjusted by taking advantage of the relative credibility preference of the pieces of evidence. Thanks to introducing the fuzzy preference relations analysis based on the belief entropy, it can automatically construct the fuzzy preference relation matrix, rather than being determined by experts, which decreases the epistemic non-determinacy in the decision-making process. Finally, the adjusted weights of the pieces of evidence are applied to modify the body of the pieces of evidence before utilizing Dempster’s combination rule. The flowchart of the proposal is shown in Figure 1.

### 3.1. Calculate the Support Degree of the Evidence

Step 1: The distance measure $d_{ij}$ between the BPAs $m_{i}$(i=1,2,…,k) and $m_{j}$(j=1,2,…,k) can be obtained by Equations (9) and (10); thus, a distance measure matrix $DMM={\left(d_{ij}\right)}_{k\times k}$ can be constructed as follows:
  (18) $$DMM=\left[\begin{array}{ccccc} 0 & \cdots & d_{1i} & \cdots & d_{1k}\\ \vdots & \cdots & \vdots & \vdots & \vdots \\ d_{i1} & \cdots & 0 & \cdots & d_{ik}\\ \vdots & \cdots & \vdots & \vdots & \vdots \\ d_{k1} & \cdots & d_{ki} & \cdots & 0 \end{array}\right].\;$$
Step 2: The similarity measure $S_{ij}$ between the BPAs $m_{i}$ and $m_{j}$ can be obtained by:
  (19) $$S_{ij}=1-d_{ij},\;1\leq i\leq k;1\leq j\leq k.$$
  Then, the similarity measure matrix $SMM={\left(S_{ij}\right)}_{k\times k}$ can be constructed as follows:
  (20) $$SMM=\left[\begin{array}{ccccc} 1 & \cdots & S_{1i} & \cdots & S_{1k}\\ \vdots & \cdots & \vdots & \vdots & \vdots \\ S_{i1} & \cdots & 1 & \cdots & S_{ik}\\ \vdots & \cdots & \vdots & \vdots & \vdots \\ S_{k1} & \cdots & S_{ki} & \cdots & 1 \end{array}\right].\;$$
Step 3: The support degree of the BPA $m_{i}$ is defined as follows:
  (21) $$Sup_{i}=\sum_{j=1,j\neq i}^{k}S_{ij},\;1\leq i\leq k.$$
Step 4: The support degree of the BPA $m_{i}$ is normalized as below, which is denoted as $\tilde{S}up_{i}$:
  (22) $$\tilde{S}up_{i}=\frac{Sup_{i}}{\sum_{r=1}^{k}Sup_{r}},\;1\leq i\leq k.$$

### 3.2. Generate the Credibility Value of the Evidence

In the course of information fusion, it is important to identify the relatively credible evidence in terms of the obtained pieces of evidence. Due to the increase of the uncertainty in the collection of information, the degree of anarchy involved in the systems rises, which violates the necessary condition to use Dempster’s rule of combination. Utilizing the ordered information can make the technologies based on the Dempster–Shafer evidence theory more robust. Therefore, we take advantage of the fuzzy preference relations analysis [79] based on the belief entropy [43] to indicate the relative credibility preference among the pieces of evidence. The concrete steps are listed as follows:

Step 1: The belief entropy of the BPA $m_{i}$(i=1,2,…,k) is calculated by leveraging Equation (11).
  Because the belief entropy of the evidence may be zero in a certain case, in order to avoid allocating zero weight to such kinds of evidence, we utilize the information volume $IV_{i}$ for measuring the uncertainty of the BPA $m_{i}$ as below:
  (23) $$IV_{i}=e^{E_{d}\left(m_{i}\right)}=e^{-\sum_{A\subseteq \Theta }m\left(A\right)\;log\;\frac{m(A)}{2^{\left|A\right|}-1}},\;1\leq i\leq k.$$
Step 2: The information volume of the BPA $m_{i}$ is normalized as below, which is denoted as $\tilde{I}V_{i}$:
  (24) $$\tilde{I}V_{i}=\frac{IV_{i}}{\sum_{r=1}^{k}IV_{r}},\;1\leq i\leq k.$$
Step 3: The fuzzy preference relation matrix $P={\left(p_{ij}\right)}_{k\times k}$, where $p_{ij}\in \left[0,1\right]$ can be constructed by the following steps:
  Step 3-1: According to Definition 6, the diagonal element $p_{ii}$ is assigned to 0.5.
  Step 3-2: If there are only two pieces of evidence, all of the off-diagonal elements $p_{ij}$ and $p_{ji}$ will be assigned to 0.5, because we have no sufficient evidence to detect how the pieces of evidence are preferred with respect to each other. Thus, the fuzzy preference relation matrix $P={\left(p_{ij}\right)}_{k\times k}$ can be constructed by:
    (25) $$P={\left(p_{ij}\right)}_{k\times k}=\left[\begin{array}{cc} 0.5 & 0.5\\ 0.5 & 0.5 \end{array}\right].\;$$
  Step 3-3: If there are more than two pieces of evidence, the variance of entropy for the BPA $m_{i}$(1≤i≤k) will be calculated as follows:
    (26) $$Var_{i}=Var\left(\left\{\tilde{I}V_{1},\tilde{I}V_{2},\ldots ,\tilde{I}V_{i-1},\tilde{I}V_{i+1},\ldots ,\tilde{I}V_{k}\right\}\right).$$
  Step 3-4: The smaller the value $Var_{i}$ has, the more conflict the evidence has in the decision-making system, so that a small preference value is supposed to be assigned to this evidence. Otherwise, the bigger the value $Var_{i}$ has, the less conflict the evidence has in the decision-making system, so that a big preference value is supposed to be assigned to this evidence. On the basis of the above variance of entropy, the off-diagonal elements $p_{ij}$ and $p_{ji}$ will be computed by Equations (27) and (28) introduced in [79].
    (27) $$\begin{array}{cc} p_{ij} & =\frac{Var_{i}}{Var_{i}+Var_{j}}, \end{array}$$
    (28) $$\begin{array}{cc} p_{ji} & =\frac{Var_{j}}{Var_{i}+Var_{j}}, \end{array}$$
    where 1≤i≤k and 1≤j≤k.
Step 4: Based on the obtained fuzzy preference relation matrix $P={\left(p_{ij}\right)}_{k\times k}$, the consistency matrix $\bar{P}$ can be constructed by Equation (16).
Step 5: With the consistency matrix $\bar{P}$, the credibility value of the BPA $m_{i}$ is defined based on Equation (17):
  (29) $$Crd_{i}=\frac{2}{k^{2}}\sum_{j=1}^{k}{\bar{p}}_{ij},\;1\leq i\leq k.$$
  We can notice that $\sum_{i=1}^{k}Crd_{i}=1$. Hence, the credibility value of each piece of evidence is regarded as a weight that indicates the relative credibility preference in terms of the evidence.

### 3.3. Fuse the Weighted Average Evidence

Step 1: Based on the credibility degree $Crd_{i}$, the normalized support degree of the BPA $m_{i}$ will be adjusted, denoted as $ASup_{i}$:
  (30) $$ASup_{i}=Crd_{i}\times \tilde{S}up_{i},\;1\leq i\leq k.$$
Step 2: The $ASup_{i}$ is normalized as below, denoted as $\tilde{A}Sup_{i}$, which is considered as the final weight of the BPA $m_{i}$.
  (31) $$\tilde{A}Sup_{i}=\frac{ASup_{i}}{\sum_{r=1}^{k}ASup_{r}},\;1\leq i\leq k.$$
Step 3: On the basis of the final weight $\tilde{A}Sup_{i}$, the weighted average evidence WAE(m) can be obtained as follows:
  (32) $$WAE\left(m\right)=\sum_{i=1}^{k}\left(\tilde{A}Sup_{i}\times m_{i}\right),\;1\leq i\leq k,$$
  where *k* denotes the number of BPAs and $m_{i}$ represents the *i*-th BPA, which are modeled from the sensor reports.
Step 4: The weighted average evidence WAE(m) is combined through Dempster’s combination rule, namely Equation (7), by k−1 times, if there are *k* number of pieces of evidence. Then, the final combination result of multiple pieces of evidence can be obtained.

## 4. Experiment

In this section, to demonstrate the effectiveness of the proposal, a numerical example is illustrated.

**Example** **1.** *Consider a target recognition problem based on multiple sensors associated with the sensor reports that are collected from five different types of sensors. These sensor reports that are modeled as the BPAs are given in Table 1 from [72], where the frame of discernment* Θ *that consists of three potential objects is given by Θ={A,B,C}.*

Step 1: Construct the distance measure matrix $DMM={\left(d_{ij}\right)}_{k\times k}$ as follows:
  $$DMM=\left(\begin{array}{ccccc} 0 & 0.5386 & 0.3495 & 0.3257 & 0.3311\\ 0.5386 & 0 & 0.8142 & 0.7850 & 0.7906\\ 0.3495 & 0.8142 & 0 & 0.0300 & 0.0374\\ 0.3257 & 0.7850 & 0.0300 & 0 & 0.0354\\ 0.3311 & 0.7906 & 0.0374 & 0.0354 & 0 \end{array}\right).$$
Step 2: Construct the similarity measure matrix $SMM={\left(S_{ij}\right)}_{k\times k}$ as follows:
  $$SMM=\left(\begin{array}{ccccc} 1 & 0.4614 & 0.6505 & 0.6743 & 0.6689\\ 0.4614 & 1 & 0.1858 & 0.2150 & 0.2094\\ 0.6505 & 0.1858 & 1 & 0.9700 & 0.9626\\ 0.6743 & 0.2150 & 0.9700 & 1 & 0.9646\\ 0.6689 & 0.2094 & 0.9626 & 0.9646 & 1 \end{array}\right).$$
Step 3: Calculate the support degree of the BPA $m_{i}$ as below:
  $Sup_{1}$ = 2.4551,
  $Sup_{2}$ = 1.0716,
  $Sup_{3}$ = 2.7689,
  $Sup_{4}$ = 2.8239,
  $Sup_{5}$ = 2.8055.
Step 4: Normalize the support degree of the BPA $m_{i}$ as follows:
  $\tilde{S}up_{1}$ = 0.2059,
  $\tilde{S}up_{2}$ = 0.0899,
  $\tilde{S}up_{3}$ = 0.2322,
  $\tilde{S}up_{4}$ = 0.2368,
  $\tilde{S}up_{5}$ = 0.2353.
Step 5: Measure the information volume of the BPA $m_{i}$ as below:
  $IV_{1}$ = 4.7894,
  $IV_{2}$ = 1.5984,
  $IV_{3}$ = 6.1056,
  $IV_{4}$ = 6.6286,
  $IV_{5}$ = 5.8767.
Step 6: Normalize the information volume of the BPA $m_{i}$ as follows:
  $\tilde{I}V_{1}$ = 0.1916,
  $\tilde{I}V_{2}$ = 0.0639,
  $\tilde{I}V_{3}$ = 0.2442,
  $\tilde{I}V_{4}$ = 0.2652,
  $\tilde{I}V_{5}$ = 0.2351.
Step 7: Construct the fuzzy preference relation matrix $P={\left(p_{ij}\right)}_{n\times n}$ as follows:
  $$P=\left(\begin{array}{ccccc} 0.5000 & 0.9002 & 0.5238 & 0.5559 & 0.5144\\ 0.0998 & 0.5000 & 0.1087 & 0.1219 & 0.1051\\ 0.4762 & 0.8913 & 0.5000 & 0.5323 & 0.4906\\ 0.4441 & 0.8781 & 0.4677 & 0.5000 & 0.4583\\ 0.4856 & 0.8949 & 0.5094 & 0.5417 & 0.5000 \end{array}\right).$$
Step 8: Construct the consistency matrix $\bar{P}={\left({\bar{p}}_{ik}\right)}_{n\times n}$ as follows:
  $$\bar{P}=\left(\begin{array}{ccccc} 0.5000 & 0.9117 & 0.5208 & 0.5492 & 0.5125\\ 0.0883 & 0.5000 & 0.1091 & 0.1375 & 0.1008\\ 0.4792 & 0.8909 & 0.5000 & 0.5284 & 0.4917\\ 0.4508 & 0.8625 & 0.4716 & 0.5000 & 0.4633\\ 0.4875 & 0.8992 & 0.5083 & 0.5367 & 0.5000 \end{array}\right).$$
Step 9: Calculate the credibility value of the BPA $m_{i}$ as below:
  $Crd_{1}$ = 0.2395,
  $Crd_{2}$ = 0.0749,
  $Crd_{3}$ = 0.2312,
  $Crd_{4}$ = 0.2198,
  $Crd_{5}$ = 0.2345.
Step 10: Adjust the normalized support degree of the BPA $m_{i}$ based on the credibility value as below:
  $ASup_{1}$ = 0.0493,
  $ASup_{2}$ = 0.0067,
  $ASup_{3}$ = 0.0537,
  $ASup_{4}$ = 0.0521,
  $ASup_{5}$ = 0.0552.
Step 11: Normalize the adjusted support degree of the BPA $m_{i}$ as below:
  $\tilde{A}Sup_{1}$ = 0.2273,
  $\tilde{A}Sup_{2}$ = 0.0310,
  $\tilde{A}Sup_{3}$ = 0.2474,
  $\tilde{A}Sup_{4}$ = 0.2399,
  $\tilde{A}Sup_{5}$ = 0.2543.
Step 12: Compute the weighted average evidence as below:
  m({A}) = 0.5213,
  m({B}) = 0.1606,
  m({C}) = 0.0713,
  m({A,C}) = 0.2469.
Step 13: Combine the weighted average evidence by utilizing Dempster’s rule of combination four times. The results of the combination for the first time are shown below:
  m({A}) = 0.8066,
  m({B}) = 0.0393,
  m({C}) = 0.0614,
  m({A,C}) = 0.0929.
  For the combination for the second time, the results are listed as follows:
  m({A}) = 0.9239,
  m({B}) = 0.0087,
  m({C}) = 0.0362,
  m({A,C}) = 0.0317.
  Next, the results of the third combination are calculated as:
  m({A}) = 0.9701,
  m({B}) = 0.0019,
  m({C}) = 0.0184,
  m({A,C}) = 0.0105.
  Then, the combination results of the fourth time, namely the final fusing results, are produced as follows:
  m({A}) = 0.9888,
  m({B}) = 0.0004,
  m({C}) = 0.0087,
  m({A,C}) = 0.0034.

From Example 1, we can notice that the evidence $m_{2}$ highly conflicts with other pieces of evidence, because the normalized support degree of the evidence $m_{2}$ is 0.0899, which is much lower than the normalized support degrees of other pieces of evidence.

**Figure 2:**
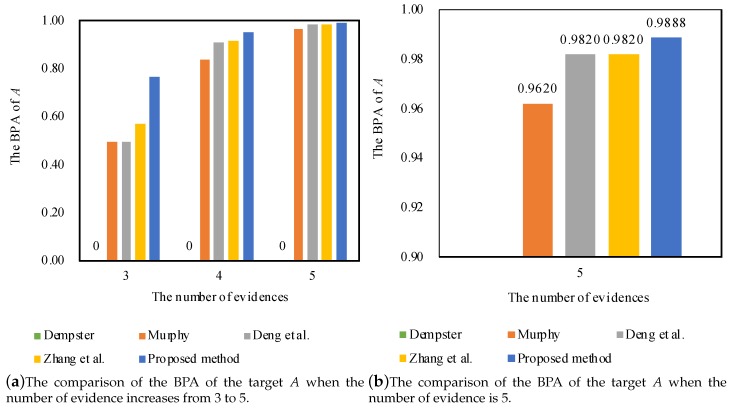
The comparison of different methods in Example 1.

The fusing results that are generated by different combination methods are shown in Table 2. The comparisons of the BPA of the target *A* by different combination rules are shown in Figure 2.

As shown in Table 2, Dempster’s combination rule generates a counterintuitive result, even though the other four pieces of evidence support the target *A*. As the number of pieces of evidence increases from 3–5, Murphy’s method [71], Deng et al.’s method [72], Zhang et al.’s method [73] and the proposed method present reasonable results. Additionally, the proposed method is efficient in dealing with the conflicting pieces of evidence with better convergence as shown in Figure 2. The reason is that the proposal not only makes use of the function of evidence distance to obtain the evidence’s support degree, but also adopts the fuzzy preference relations analysis based on the belief entropy to measure the relative credibility preference among the pieces of evidence. After considering these aspects, the unreliable evidence’s weight is decreased, so that its negative effect can be relieved on the final fusing results compared to other methods.

## 5. Application

In this section, the proposal is applied to the fault diagnosis of a motor rotor, where the practical data in [27] are used for the comparison with the related method.

### 5.1. Problem Statement

Supposing that the frame of discernment Θ, which consists of three types of faults for a motor rotor is given by Θ = {Rotorunbalance, Rotormisalignment, Pedestallooseness} = $\left\{F_{1},F_{2},F_{3}\right\}$. The set of vibration acceleration sensors given by *S* = $\left\{S_{1},S_{2},S_{3}\right\}$ is positioned at different places for gathering the vibration signals. The acceleration vibration frequency amplitudes at 1X frequency, 2X frequency and 3X frequency are considered as the fault feature variables. The collected sensor reports at 1X frequency, 2X frequency and 3X frequency that are modeled as BPAs are given in Table 3, Table 4 and Table 5, respectively, where $m_{1}\left(\cdot \right)$, $m_{2}\left(\cdot \right)$ and $m_{3}\left(\cdot \right)$ represent the BPAs reported from the three vibration acceleration sensors $S_{1}$, $S_{2}$ and $S_{3}$.

### 5.2. Motor Rotor Fault Diagnosis Based on the Proposed Method

#### 5.2.1. Motor Rotor Fault Diagnosis at 1X Frequency

According to the proposed method in Section 3 and Table 3’s BPAs modeled by the collected sensor reports at the frequency of 1X, the weighted average evidence in terms of motor rotor fault diagnosis at 1X frequency is obtained as follows:

$m(\left\{F_{2}\right\})$ = 0.5636,
$m(\left\{F_{3}\right\})$ = 0.0006,
$m(\left\{F_{1},F_{2}\right\})$ = 0.0782,
$m(\left\{F_{1},F_{2},F_{3}\right\})$ = 0.3576.

After that, the weighted average evidence in terms of motor rotor fault diagnosis at 1X frequency is fused by utilizing Dempster’s rule of combination two times. The results of the combination for the first time are shown below:

$m(\left\{F_{2}\right\})$ = 0.8095,
$m(\left\{F_{3}\right\})$ = 0.0004,
$m(\left\{F_{1},F_{2}\right\})$ = 0.0621,
$m(\left\{F_{1},F_{2},F_{3}\right\})$ = 0.1280.

Then, the results of the combination for the second time, namely the final fusing results for motor rotor fault diagnosis at 1X frequency, are generated as follows:

$m(\left\{F_{2}\right\})$ = 0.9169,
$m(\left\{F_{3}\right\})$ = 0.0002,
$m(\left\{F_{1},F_{2}\right\})$ = 0.0371,
$m(\left\{F_{1},F_{2},F_{3}\right\})$ = 0.0458.

#### 5.2.2. Motor Rotor Fault Diagnosis at 2X Frequency

On the basis of the proposed method in Section 3 and Table 4’s BPAs modeled by the collected sensor reports at the frequency of 2X, the weighted average evidence with respect to motor rotor fault diagnosis at 2X frequency is obtained as follows:

$m(\left\{F_{2}\right\})$ = 0.7754,
$m(\left\{F_{1},F_{2},F_{3}\right\})$ = 0.2246.

Next, by leveraging Dempster’s rule of combination, the weighted average evidence with respect to motor rotor fault diagnosis at 2X frequency is fused two times. For the combination for the first time, the fusion results are given below:

$m(\left\{F_{2}\right\})$ = 0.9496,
$m(\left\{F_{1},F_{2},F_{3}\right\})$ = 0.0504.

Afterwards, for the combination for the second time, the final fusion results with respect to motor rotor fault diagnosis at 2X frequency are shown below:

$m(\left\{F_{2}\right\})$ = 0.9887,
$m(\left\{F_{1},F_{2},F_{3}\right\})$ = 0.0113.

#### 5.2.3. Motor Rotor Fault Diagnosis at 3X Frequency

By applying the proposed method in Section 3 and Table 5’s BPAs modeled by the collected sensor reports at the frequency of 3X, the weighted average evidence with regard to motor rotor fault diagnosis at 3X frequency is obtained as follows:

$m(\left\{F_{1}\right\})$ = 0.3028,
$m(\left\{F_{2}\right\})$ = 0.4323,
$m(\left\{F_{1},F_{2}\right\})$ = 0.2254,
$m(\left\{F_{1},F_{2},F_{3}\right\})$ = 0.0395.

Therewith, the weighted average evidence with regard to motor rotor fault diagnosis at 3X frequency is fused by utilizing Dempster’s rule of combination two times. The combination results for the first time are listed below:

$m(\left\{F_{1}\right\})$ = 0.3415,
$m(\left\{F_{2}\right\})$ = 0.5634,
$m(\left\{F_{1},F_{2}\right\})$ = 0.0929,
$m(\left\{F_{1},F_{2},F_{3}\right\})$ = 0.0021.

Then, the final combination results for the second time for motor rotor fault diagnosis at 3X frequency are shown below:

$m(\left\{F_{1}\right\})$ = 0.3266,
$m(\left\{F_{2}\right\})$ = 0.6365,
$m(\left\{F_{1},F_{2}\right\})$ = 0.0368,
$m(\left\{F_{1},F_{2},F_{3}\right\})$ = 0.0001.

### 5.3. Discussion

According to the results as shown in Table 6, Table 7 and Table 8, we can notice that the proposed method can diagnose the fault type $F_{2}$, which is consistent with Jiang et al.’s method [27]. Even facing the conflicting sensor reports where the normalized support degrees of the sensor reports are different at 1X frequency, 2X frequency and 3X frequency, both of the methods can well manage the conflicting pieces of evidence and diagnose the fault type $F_{2}$.

**Figure 3:**
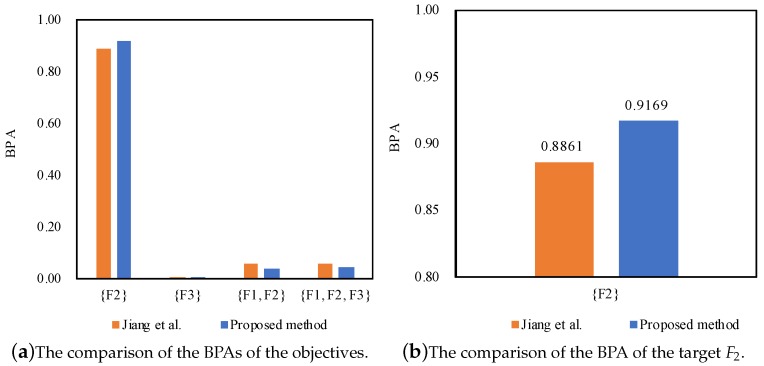
The comparison of different methods for motor rotor fault diagnosis at 1X frequency.

**Figure 4:**
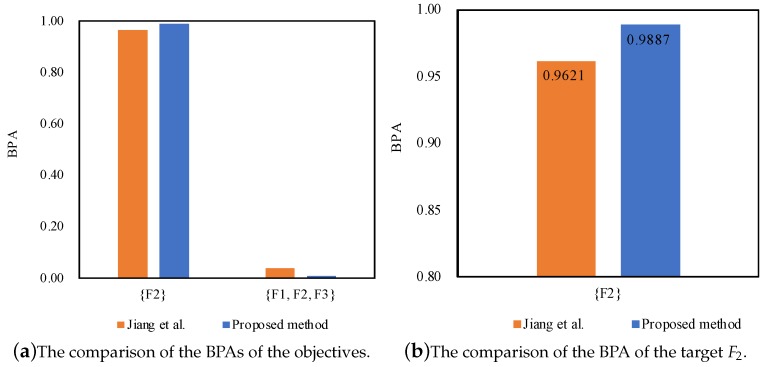
The comparison of different methods for motor rotor fault diagnosis at 2X frequency.

**Figure 5:**
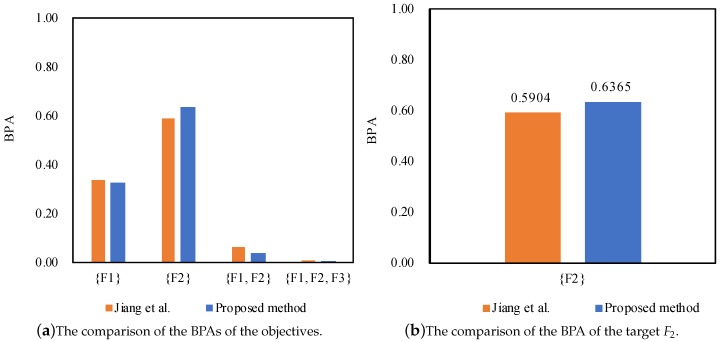
The comparison of different methods for motor rotor fault diagnosis at 3X frequency.

Furthermore, the proposed method outperforms Jiang et al.’s method [27] in terms of dealing with the conflicting pieces of evidence, as well as coping with the uncertainty as shown in Figure 3, Figure 4 and Figure 5, because the belief degrees assigned to the target $F_{2}$ at 1X frequency, 2X frequency and 3X frequency by the proposed method rise to 91.69%, 98.87% and 63.65%, respectively, while the belief degrees assigned to the target $F_{2}$ at 1X frequency, 2X frequency and 3X frequency by the method Jiang et al. [27] are 88.61%, 96.21% and 59.04%, respectively.

On the other hand, the uncertainty $\left\{F_{1},F_{2}\right\}$ falls to 0.0371 from 0.0582, and the uncertainty $\left\{F_{1},F_{2},F_{3}\right\}$ falls to 0.0458 from 0.0555 at 1X frequency; the uncertainty $\left\{F_{1},F_{2},F_{3}\right\}$ drops to 0.0113 from 0.0371 at 2X frequency; the uncertainty $\left\{F_{1},F_{2}\right\}$ falls to 0.0368 from 0.0651, and the uncertainty $\left\{F_{1},F_{2},F_{3}\right\}$ falls to 0.0001 from 0.0061 at 3X frequency. The main reason is that the proposed method not only takes the support degree of the sensor reports into account by making use of the function of evidence distance, but also considers the relative credibility preference of the sensor reports by taking advantage of the fuzzy preference relations analysis on the basis of the belief entropy. As a result, the proposed method can diagnose motor rotor fault more accurately.

## 6. Conclusions

In this paper, on account of the support degree among the pieces of evidence, the uncertainty measure of the evidence and the effect of the relative credibility of evidence on the weight, a novel method for multi-sensor data fusion was proposed. The proposed method was a hybrid methodology by integrating the distance of evidence, belief entropy and fuzzy preference relation analysis. It consisted of three main procedures. Firstly, the support degree of the evidence was calculated to represent the reliability of the evidence. Secondly, the credibility value of the evidence was generated to indicate the relative credibility preference of the evidence. Thirdly, based on the first two procedures, the weighted average evidence was obtained; thus, it could be fused by applying Dempster’s combination rule. As described above, the proposed method was a kind of approach to pre-process the bodies of evidence. Through a numerical example, it was illustrated that the proposal was more effective and feasible than other related methods to handle the conflicting evidence combination problem under a multi-sensor environment with better convergence. On the other hand, a practical application in fault diagnosis was presented to demonstrate that the proposed method could diagnose the faults more accurately.

In future work, I intend to consider further fault diagnosis of complicated equipment/systems that involves certain faults, such as cracks and misalignment. On the other hand, multiple faults, like bearing faults, rotor-related faults, etc., will be taken into account in future work to improve the robustness of the technique.

References

## Figures and Tables

**Table 1:** The basic probability assignments (BPAs) for Example 1.

BPA	{A}	{B}	{C}	{A,C}
$S_{1}:m_{1}\left(\cdot \right)$	0.41	0.29	0.30	0.00
$S_{2}:m_{2}\left(\cdot \right)$	0.00	0.90	0.10	0.00
$S_{3}:m_{3}\left(\cdot \right)$	0.58	0.07	0.00	0.35
$S_{4}:m_{4}\left(\cdot \right)$	0.55	0.10	0.00	0.35
$S_{5}:m_{5}\left(\cdot \right)$	0.60	0.10	0.00	0.30

**Table 2:** Combination results of the evidence in terms of different combination rules.

Evidence	Method	{A}	{B}	{C}	{AC}	Target
$m_{1},m_{2},m_{3}$	Dempster [23]	0	0.6350	0.3650	0	B
	Murphy [71]	0.4939	0.4180	0.0792	0.0090	A
	Deng et al. [72]	0.4974	0.4054	0.0888	0.0084	A
	Zhang et al. [73]	0.5681	0.3319	0.0929	0.0084	A
	Proposed method	0.7617	0.1127	0.1176	0.0080	A
$m_{1},m_{2},m_{3},m_{4}$	Dempster [23]	0	0.3321	0.6679	0	C
	Murphy [71]	0.8362	0.1147	0.0410	0.0081	A
	Deng et al. [72]	0.9089	0.0444	0.0379	0.0089	A
	Zhang et al. [73]	0.9142	0.0395	0.0399	0.0083	A
	Proposed method	0.9507	0.0060	0.0334	0.0087	A
$m_{1},m_{2},m_{3},m_{4},m_{5}$	Dempster [23]	0	0.1422	0.8578	0	C
	Murphy [71]	0.9620	0.0210	0.0138	0.0032	A
	Deng et al. [72]	0.9820	0.0039	0.0107	0.0034	A
	Zhang et al. [73]	0.9820	0.0034	0.0115	0.0032	A
	Proposed method	0.9888	0.0004	0.0087	0.0034	A

**Table 3:** The collected sensor reports at the frequency of 1X modeled as BPAs.

BPA	$\left\{F_{2}\right\}$	$\left\{F_{3}\right\}$	$\left\{F_{1},F_{2}\right\}$	$\left\{F_{1},F_{2},F_{3}\right\}$
$S_{1}:m_{1}\left(\cdot \right)$	0.8176	0.0003	0.1553	0.0268
$S_{2}:m_{2}\left(\cdot \right)$	0.5658	0.0009	0.0646	0.3687
$S_{3}:m_{3}\left(\cdot \right)$	0.2403	0.0004	0.0141	0.7452

**Table 4:** The collected sensor reports at the frequency of 2X modeled as BPAs.

BPA	$\left\{F_{2}\right\}$	$\left\{F_{1},F_{2},F_{3}\right\}$
$S_{1}:m_{1}\left(\cdot \right)$	0.6229	0.3771
$S_{2}:m_{2}\left(\cdot \right)$	0.7660	0.2341
$S_{3}:m_{3}\left(\cdot \right)$	0.8598	0.1402

**Table 5:** The collected sensor reports at the frequency of 3X modeled as BPAs.

BPA	$\left\{F_{1}\right\}$	$\left\{F_{2}\right\}$	$\left\{F_{1},F_{2}\right\}$	$\left\{F_{1},F_{2},F_{3}\right\}$
$S_{1}:m_{1}\left(\cdot \right)$	0.3666	0.4563	0.1185	0.0586
$S_{2}:m_{2}\left(\cdot \right)$	0.2793	0.4151	0.2652	0.0404
$S_{3}:m_{3}\left(\cdot \right)$	0.2897	0.4331	0.2470	0.0302

**Table 6:** Fusion results of different methods for motor rotor fault diagnosis at 1X frequency.

Method	$\left\{F_{2}\right\}$	$\left\{F_{3}\right\}$	$\left\{F_{1},F_{2}\right\}$	$\left\{F_{1},F_{2},F_{3}\right\}$	Target
Jiang et al. [27]	0.8861	0.0002	0.0582	0.0555	$F_{2}$
Proposed method	0.9169	0.0002	0.0371	0.0458	$F_{2}$

**Table 7:** Fusion results of different methods for motor rotor fault diagnosis at 2X frequency.

Method	$\left\{F_{2}\right\}$	$\left\{F_{1},F_{2},F_{3}\right\}$	Target
Jiang et al. [27]	0.9621	0.0371	$F_{2}$
Proposed method	0.9887	0.0113	$F_{2}$

**Table 8:** Fusion results of different methods for motor rotor fault diagnosis at 3X frequency.

Method	$\left\{F_{1}\right\}$	$\left\{F_{2}\right\}$	$\left\{F_{1},F_{2}\right\}$	$\left\{F_{1},F_{2},F_{3}\right\}$	Target
Jiang et al. [27]	0.3384	0.5904	0.0651	0.0061	$F_{2}$
Proposed method	0.3266	0.6365	0.0368	0.0001	$F_{2}$
